# Approach to Design and Evaluate Digital Tools to Enhance Young Adult Participation in Clinical Trials: Co-Design and Controlled Intercept Study

**DOI:** 10.2196/70852

**Published:** 2025-04-11

**Authors:** Tim Mackey, Raphael E Cuomo, Qing Xu, Tiana J McMann, Zhuoran Li, Mingxiang Cai, Christine Wenzel, Joshua S Yang

**Affiliations:** 1 Global Health Program Department of Anthropology University of California San Diego La Jolla, CA United States; 2 Global Health Policy and Data Institute San Diego, CA United States; 3 S-3 Research San Diego, CA United States; 4 Department of Anesthesiology School of Medicine University of California San Diego San Diego, CA United States; 5 Department of Public Health California State University, Fullerton Fullerton, CA United States

**Keywords:** health, clinical trials, COVID-19, digital health, coronavirus disease

## Abstract

**Background:**

Certain populations are underrepresented in clinical trials, limiting the generalizability of new treatments and their efficacy and uptake in these populations. It is essential to identify and understand effective strategies for enrolling young adults in clinical trials, as they represent a vital and key demographic for future clinical trial participation.

**Objective:**

This study aimed to develop, test, and evaluate digital tools designed to encourage the participation of young adults in the clinical trial process. An interdisciplinary approach, incorporating social listening, qualitative focus groups, and co-design workshops, was used to achieve this goal.

**Methods:**

Digital tools were designed and evaluated using a 4-phase approach that included: (1) social listening to characterize lived experiences with COVID-19 trials as self-reported by online users, (2) qualitative focus groups with young adults to explore specific lived attitudes and experiences related to COVID-19 clinical research hesitancy and engagement, (3) a series of cocreation and co-design workshops to build digital tools aimed at encouraging clinical trial participation, and (4) a controlled intercept study to assess the usability and specific outcome measures of the co-designed digital tools among young adults.

**Results:**

A significantly higher change in the likelihood of participating in a clinical trial post exposure was observed among study participants when exposed to prototypes of a mobile app (Δ=0.74 on a 10-point scale, *P*<.01) and website (Δ=0.93, *P*<.01) compared to those exposed to a Facebook ad (Δ=0.21) but not a digital flyer (Δ=0.58). Furthermore, those exposed to the mobile app (x̅=5.76, *P*=.04) and electronic flier (x̅=5.72, *P*=.04), but not the website (x̅=5.55), exhibited significantly higher postexposure interest in learning about clinical trials when compared to participants exposed to the Facebook (Meta) ad (x̅=5.06). Participants in the intercept study were more likely to consider joining a clinical trial after seeing a mobile app (Δ=0.74, *P*<.01) or website (Δ=0.93, *P*<.001) compared to a Facebook ad (Δ=0.21), but the digital flyer (Δ=0.58) did not show a significant difference. In addition, those who saw the mobile app (x̅=5.76, *P*=.04) or the digital flyer (x̅=5.72, *P*=.04) showed more interest in learning about clinical trials than those who saw the Facebook ad (x̅=5.06), though the website (x̅= 5.55) did not significantly impact interest.

**Conclusions:**

Mobile apps and web pages co-designed with young diverse adults may represent effective digital tools to advance shared goals of encouraging inclusive clinical trials.

## Introduction

It is essential to identify and understand effective strategies for enrolling young adults in clinical trials, as they represent a vital and key demographic for future clinical trial participation [1-3]. However, young adult enrollment in clinical trials varies by study and institution type but generally occurs at lower rates relative to children and older adult demographics [4,5]. Furthermore, the most recent National Institutes of Health data on age at enrollment in clinical research reported that 18-34-year-olds accounted for 19.5% of enrollment in Fiscal Year 2021 [6]. Young adults have distinctly different interests, values, and reasons for participating that need to be purposefully integrated into trial recruitment, participation, and retention practices [7-10]. Increasing participation in all types of clinical trials remains critical to ensuring the generalizability of new treatments and their efficacy and uptake in specific populations, including among young adults [11]. Further, clinical trial sites may lack experience, or the tools needed to recruit young adults, especially racial and ethnic minority populations [12].

While previous studies have examined the utility of mobile apps, social media, e-consenting tools, blockchain technology, web-based programs, and online messaging to improve clinical trial enrollment, few have specifically addressed the unique barriers faced by the young adult population. Yet, digital modalities have grown in popularity among this population for many indications including disease interventions and browsing emerging health information [13-15]. Furthermore, 62% of young adults aged 18-29 years report using the internet daily or almost constantly [16]. However, a recent review article that examined the medical, engineering, and computer science literature on this topic identified different forms of technology and diseases addressed by digital tools designed to encourage participation in clinical trials but did not specifically report results for young adult-aged demographics [10].

To fill this critical gap, the objective of this study was to use social listening, qualitative focus groups, and co-design workshops to develop, test, and evaluate digital tools purposefully designed to encourage young adults to participate in the clinical trial process. To generate outcome measures for these tools, a controlled intercept study assessed whether the tool increased the propensity to enroll in clinical trials among young adults.

## Methods

### Overview

**Figure 1:**
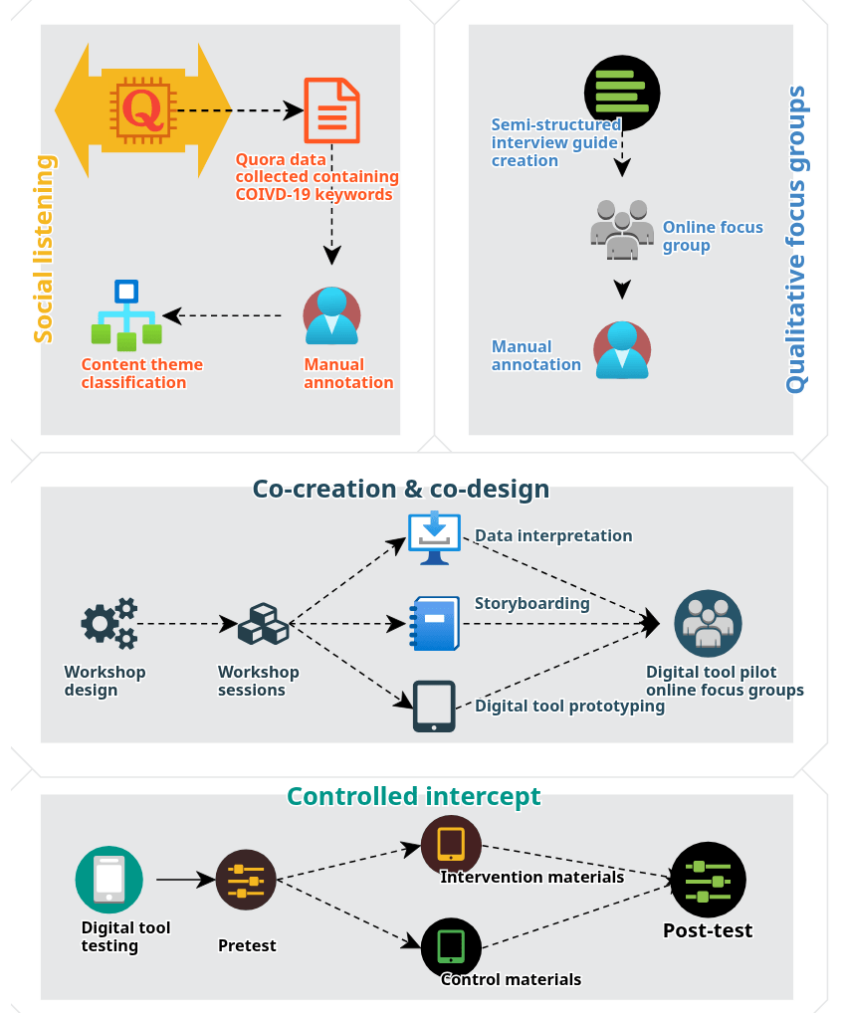
Summary of 4-step study methodology including social listening, qualitative focus groups among young adults, cocreation and co-design of the digital tools, and the controlled digital tool testing through intercept.

This study employed a multiphase methodology consisting of four key stages: (1) social listening to characterize lived experiences with COVID-19 trials as self-reported by users on social media; (2) qualitative focus groups with young adults in the Los Angeles-Long-Beach-Anaheim Metropolitan Statistical Area to explore cultural norms, lived experiences, and factors contributing to COVID-19 clinical research hesitancy and engagement; (3) a series of co-design workshops with diverse young adults to build digital tools aimed at encouraging clinical trial participation; and (4) a controlled intercept study conducted at a Minority Serving Institution (MSI)-designated university to assess the usability and specific outcome measures of the co-designed digital tools (refer to Multimedia Appendix 1 for additional details). A visual summary of the methodology is provided in Figure 1. This study followed the reporting guidelines for Standards for Reporting Qualitative Research for the qualitative component and the Strengthening the Reporting of Observational Studies in Epidemiology for the controlled intercept component of this study [17].

### Social Listening

To understand public discourse on COVID-19 clinical trial participation and hesitancy, we conducted social listening on Quora (a social question-and-answer platform) by filtering for keywords and hashtags (#) associated with COVID-19 clinical research terms, specific trials, vaccines, and general COVID-19 conversations (refer to Textbox 1). Quora was selected for social listening analysis based on preliminary manual searches on the platform that identified active conversations about COVID-19 trial attitudes, experiences, and other interactions on the platform discussing lived experiences associated with COVID-19 trials.

Qualitative analysis using an inductive content coding approach was used to characterize experiences, attitudes, and barriers to COVID-19 trials from social media data. Initial codes were derived from an adapted version of the COVID-19 objective and subjective factors of clinical trial participation outline for characteristics of interest from the center for information and study on clinical research participation (Table 1) [18].

**List of COVID-19 vaccine–related and clinical trial–related keywords used in Quora social listening aim of the study.**

**COVID-19 vaccine–related keywords**

- “COVID-19 vaccine”,
- “COVID 19 vaccine”,
- “SARS-CoV2 vaccine”,
- “Coronavirus disease 2019 vaccine”,
- “mRNA-1273”,
- “Pfizer; BioNTech”,
- “BNT162”,
- “Moderna”,
- “J&J”
- “Johnson & Johnson”

**Clinical trial–related keywords**

- “Clinical trial”,
- “trial”

**Table 1:** Adapted inductive content coding approach of objective and subjective factors for clinical trial participation used in social listening analysis on Quora. Codes include sources of knowledge, clinical trial concerns, and patient experiences with applicable subcodes and descriptions of each theme.

Parent code	Applicable subcodes	Description
Sources of knowledge	Physician involvement Educational programs Internet-based information Possible misinformation about trials Familial influence	Primary care providers promoting clinical trials as a treatment option; participant experiences posted via social media; family members not trusting clinical trials; false news articles via social media and other internet platforms
Clinical trial concerns	Reasons to participate Trial design (blinding, randomization) Safety concerns Regulatory considerations (eg, approval status, indication) Biopharmaceutical company history	Underrepresented populations; misconceptions about clinical trial timelines; past information about the company’s history in clinical trials; confusion regarding misreported adverse events; possibility of adverse effects; unsure if receiving placebo or experimental drug
Participant Experiences	Specific barriers to access trial^a^ Reporting of side effects^a^ Trial inclusion or exclusion criteria^a^ Suspicion based on cultural context^a^ Concerns about costs or compensation^a^ Feelings of underrepresentation^a^	Associated costs, distance, or lack of compensation for trial; trial participants sharing pre- and post-injection experiences; historical mistrust with clinical trials; uncertainty surrounding meeting inclusion or exclusion criteria; concerns regarding lack of minority representation; stopping trial to receive approved treatment

^a^Theme specific to equity in clinical research.

### Qualitative Focus Groups

Informed by themes generated in the social listening phase of this study, we conducted 16 qualitative focus groups with a convenience sample of young adults recruited through a market panel; an online platform was used to conduct focus groups to minimize barriers to participation. A semistructured protocol was used to guide phenomenologically-based focus group sessions with probes exploring themes including (1) knowledge, attitudes, and perception of clinical trials; (2) knowledge and attitudes regarding COVID-19 vaccine development and regulatory decision-making; (3) attitudes toward COVID-19 trials participation, with a focus on barriers to participation; (4) use of digital tools for research participation; and (5) factors that would increase willingness to participate in trials (refer to Multimedia Appendix 1). Focus group transcripts were coded by a group of 3 coders using ATLAS.ti 9 (Lumivero). One study author (JSY) created a preliminary codebook which was reviewed and applied to a subset of 2 transcripts by 2 coders. All coders reviewed coded transcripts line-by-line to clarify and revise codes as needed. Once a final codebook was created, 2 coders coded a full transcript until intercoder agreement of Krippendorff α>.800 at a .05 level of statistical significance was reached. Coders then separately coded the remaining transcripts. Transcripts with coding by each coder were then merged and a third coder (JSY) reconciled disagreements in coding.

### Co-Design Workshops and Usability Testing

After data were generated from social listening and focus groups, a series of co-design workshops were held with a group of students at a large, public MSI-designated university, to design digital tools aimed at encouraging racial and ethnic minority young adults to participate in clinical research. The human-centered design approach and activities introduced by IDEO.org were used to guide the design process [19].

The first session of the co-design approach presented findings from social listening and focus groups to frame the challenge of clinical trial participation among diverse young adults. A discussion about how participants interpret the data based upon their own experiences and those of their family, friends, and communities and associated barriers to trial participation was fostered using exercises such as insight statements, “How Might We?,” brainstorming, and rapid prototype activities. The research team took outputs from participants and created a mock-up of a potential digital tool that was used as the basis for a second cocreation session; the mock-up was refined through group interviews, “Ways to Grow,” and additional brainstorming activities. After continued refinement, a third co-design session was held for direct feedback and role-playing with the digital tools.

The research team used co-design session data and digital health best practices principles in iteratively creating prototypes of the digital tools into an interactive mobile app wireframe using the application Justinmind and a website tool using webflow. In developing prototypes of digital tools, an emphasis was placed on incorporating digital health principles into design. The digital health principles included (1) identification of unmet needs in the context of clinical trial information and engagement; (2) identifying the target audience values, benefits, and needs; (3) addressing accessibility issues (eg, platform and delivery preferences); and (4) assessing challenges to scalability.

After prototypes were refined with continuous co-design workshop input and incorporation of digital health principles, usability testing was conducted in 8 online focus groups with young adults from diverse backgrounds. After a period of digital tool interaction during focus groups, the discussion focused on assessing (1) language choice, readability, and understandability; (2) messaging and value proposition communicated in the tool; (3) cultural appropriateness of the tool; (4) UI/UX features; (5) attitudes regarding acceptance and adoption; and (6) overall usability and accessibility [20]. A semistructured protocol was used to guide focus group sessions with probes to allow for the exploration of group-specific themes. Participants were provided with a URL to interact with the digital tools during focus groups. After a period of digital tool interaction, discussion explored multiple elements of usability and final versions of the tools were developed [20].

### Intercept Study

**Figure 2:**
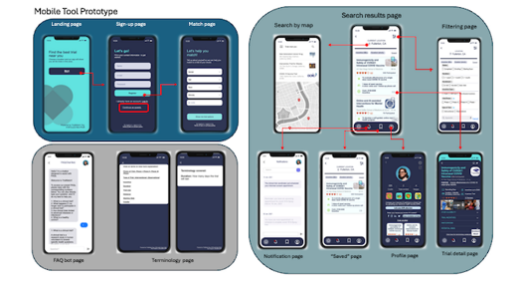
Screenshots of co-designed digital tools (mobile tool prototype).

**Figure 3:**
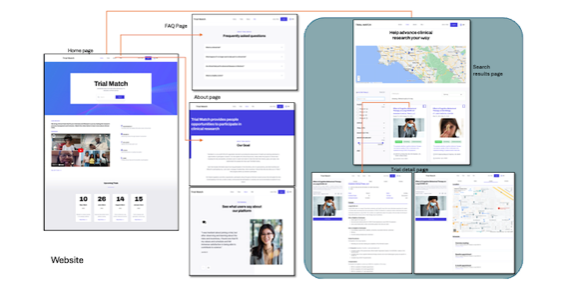
Screenshots of co-designed digital tools (website tool).

Digital tool effectiveness (Figure 2 and Figure 3) was evaluated using a controlled cross-sectional intercept study to assess the influence of digital tool exposure on the likelihood of participating in a clinical trial and interest in learning more about clinical trials. Four research assistants (RAs) were positioned along strategic high-traffic pedestrian thoroughfares and gathering places across the MSI university campus (such as the student union, restaurants, and outdoor study areas) to intercept students, explain the purposes of the study and ask them to participate, which was incentivized by an opportunity drawing. Students were included if they were 18-29 years of age.

Each RA collected data on a WiFi-connected tablet and a Qualtrics survey, which randomly assigned students to view 1 of 4 exposures: 2 of the exposures acted as controls (either a traditional clinical trial recruitment flyer in digital format or a mock Facebook ad for clinical trial recruitment, each with 3 versions of a clinical trial: 1 for post–COVID-19 condition, one for allergies, or one for diabetes) and 2 additional exposures acting as interventions (links to either functioning versions of the co-design mobile app wireframe or website).

Participants were asked to complete pretest questions about their propensity to enroll in a clinical trial via an electronic questionnaire on the tablet, then randomly assigned to view, and interact with or simply view one exposure. Upon completion, they were asked posttest questions about their propensity to participate in a clinical trial after exposure and whether, after viewing the material, how much more interested they were in learning about clinical trials. Demographic data on race, ethnicity, sex, sexual orientation, major, age, parental educational attainment, and household income was collected along with the user time spent on each exposure (refer to Multimedia Appendix 1).

The primary outcome variables were propensity to enroll in a clinical trial and whether viewing material led to greater interest in learning more about clinical trials, both measured on a 10-point scale with the lowest extreme described as “Not Likely,” the mid-point described as “Moderately Likely,” and the highest extreme described as “Very Likely.” The primary outcome was the pre-post difference between treatment and control arms, with a maximum continuous range of 1-10.

A power calculation using a 2-tailed independent samples *t* test was conducted to ascertain the minimum surveyed sample size needed to detect statistically significant differences between control and intervention arms when assuming a minor 0.25 increase in enrollment propensity in the control arm and a full additional point (x̄=1.25) increase in enrollment propensity in the intervention arm, with appreciable variation (σ=1.5 for both arms). For the power calculation, β was set to 80% and α to .05 per convention. With these parameters, a statistically significant difference would be observable with a minimum of n=74 total participants, evenly allocated to both arms. Power calculations for this study were conducted using G*Power software (Erdfelder, Faul, and Buchner).

To test the hypothesis that there existed a significant difference in pre-post change in enrollment propensity between arms, a 2-tailed independent samples *t* test was used. Multivariable linear regression was used to control potential confounders of the treatment effect, importantly including race and ethnicity. Data management and statistical analysis were carried out using IBM SPSS Statistics software.

User time spent on the mobile app, website, mock clinical trial recruitment flier, and mock Facebook trial recruitment ad exposure was recorded using Hotjar (mobile app) and Mouseflow (for website, flier, and Facebook ad). Engagement time was generated by Mouseflow for the website, flier, and Facebook ad but not for the mobile app as this metric was not available from Hotjar. Engagement time is described as the average engagement time that accounts for the duration of user activity minus inactivity for a website or page. Data collection, management, and analysis of these data were conducted using the Python and SQL programming languages.

### Ethical Considerations

The social listening phase of this study was reviewed and approved as exempt by the WCG institutional review board (IRB; WCG IRB #1-1549015-1).

For qualitative focus groups, potential participants were emailed a URL to an electronic informed consent form hosted on Qualtrics XM for completion before the conduct of focus groups. Informed consent was monitored on the Qualtrics XM project dashboard until 2 hours before each focus group; individuals who did not consent to participate were not allowed into the focus group online room. All data were collected confidentially using study aliases assigned at the beginning of focus groups with informed consent information kept separate from study data with no linking information. Participants were provided with a US $150 incentive for participation in focus groups.

For co-design sessions, discussion themes were aggregated onto poster boards, notecards, and other media used to solicit user feedback. This was not considered human subjects research and exempt from ethics approval. All information was gathered anonymously, and no compensation was provided for participation.

For the intercept study, an electronic informed consent form was programmed into the pre- and postexposure survey before any collection of data. Those who did not consent to participate were not allowed to continue the survey. Upon completion of data collection, informed consent information was deleted from the analytic data set downloaded from Qualtrics XM and analyzed anonymously. All respondents were entered into an opportunity drawing for one of 4 gift cards in the amount of US $50 as an incentive for participation.

The study methodology was approved by the institutional review board of California State University, Fullerton (IRB HSR-21-22-309).

Figure 2 shows a mobile app digital tool cocreated with young adult participants that has features to match users to a clinical trial that meets their search parameters. Also includes a FAQs (frequently asked questions) chatbot, map searching functions, a user profile page, and detailed information about a clinical trial site.

Figure 3 shows website digital tool co-created with young adult participants that has a home page to provide information about resources to search for and match to a clinical trial, map search functions, detailed information about clinical trials, a FAQ page, and mock user testimonials.

## Results

### Social Listening

Social listening was conducted to analyze user-generated posts and comments from Quora (a social question-and-answer website). Quora was chosen based on manual searches conducted by the study team across multiple social media platforms, which led to the detection of structured topics from Quora online users discussing COVID-19 clinical trial experiences, attitudes, and concerns. The findings from this analysis, published in a 2024 study, informed subsequent qualitative focus groups and co-design activities, identifying key themes related to challenges and barriers in clinical trial participation [21].

### Qualitative Focus Groups

In focus groups with 158 young adults (Asian: n=41; Black or African American: n=39; Hispanic: n=39; and White: n=39), key findings regarding existing attitudes and preferences were inductively identified. Social media was the main source of exposure to information about clinical trials, which also included other forms of media (eg, movies, and TV shows), family and friends, and school. While most participants viewed clinical trials as beneficial for society, female Black or African American participants expressed more reservations. However, all young adult study participants had low interest in participating in clinical trials. Incentives for participation were an expected component of participation, which was weighed against risk for and nature of potential side effects. Participants emphasized the importance of being provided with what they felt was transparent information and clinical trials being held as legitimate scientific endeavors without political or commercial motives. They did not want to be unduly influenced by clinical trial decisions, instead expressing confidence in their own ability to gather information and make appropriate judgments about health care decisions.

### Co-Design Workshops

Over 3 co-design sessions with an average of 20 participants per session (23, 19, and 17 participants in sessions 1, 2, and 3, respectively) that were majority female (73.9%, 17/23; 73.7%, 1419; and 70.6%, 12/17) and racial and ethnic minority participants (69.6%, 16/23; 57.9%, 11/19; and 58.9%, 10/17), 6 key features were identified as providing legitimacy and trustworthiness to a clinical trial digital tool. Personal agency was seen as an important element where the end user should be able to control aspects of the selection and enrollment process. Additional features of importance included emphasizing the purpose and mission of the trial on the landing page, the inclusion of indicators of trust within the tool (ie, tool sponsored by a nonprofit company rather than a pharmaceutical company), filters to identify key characteristics for best-fit clinical trials to participant interests, the ability to match and follow trials to receive updates and view progress, and a risk indicator to quantify a level of inherent risk for potential participants. Follow-up initial usability testing with focus groups that evaluated the digital tools suggested that mobile apps and websites can be designed to increase interest in participating in clinical trials.

### Intercept Study

A total of 351 respondents participated in the intercept study. The sample was 63.9% (223/351) female. A majority of participants 64.7% (227/351) identified as Hispanic or Latino people, 10.8% (28/351) identified as White, 15.7% (55/351) identified as Asian, 3.1% (11/351) identified as Black or African American people, 0.3% (1/351) identified as American Indian or Alaska Native, 0.3% (1/351) identified as Native Hawaiian or Other Pacific Islander people, and 4.6% (16/351) were multiracial people. No significant difference in baseline likelihood of clinical trial participation was observed between groups. However, the mobile app, website, and electronic recruitment flyer all exhibited significantly higher change in the likelihood of participating in a clinical trial post exposure when compared to participants who were exposed to the Facebook ad (refer to Table 2). Furthermore, the mobile app and electronic flier, but not the website, exhibited significantly higher postexposure interest in learning about clinical trials when compared to participants exposed to the Facebook ad. Analysis stratified by baseline likelihood of clinical trial participation found that pre-post change in participation likelihood was consistently inversely associated with baseline likelihood (refer to Table 3). A negative effect on participation likelihood was observed in the group with the highest baseline likelihood (at least 7 on a 1-10 scale), though the app and website were least likely to detract from likelihood in this subgroup. Using user tracking software Hotjar and Mouseflow, we observed that the mean time spent on the mobile app (59.65 seconds) and web-based tool (47.98 seconds) was longer than time spent on the flyer or Facebook ad (21.93 seconds and 32.62 seconds, respectively). In addition, the engagement time of the web-based tool (31.72 seconds) was longer than on the flyer or the Facebook ad (12.69 seconds and 12.97 seconds, respectively; Multimedia Appendix 1).

**Table 2:** For all intervention and control groups, baseline and change measures of primary end point (trial participation likelihood) and postintervention measure of secondary end point (trial interest), with superscripts denoting statistically significant differences between groups.

	Baseline likelihood of participating in a clinical trial (1-10 scale)	Pre-post change in the likelihood of participating in a clinical trial	Post-intervention interest in learning about clinical trials (1-10 scale)
Mobile app	4.64	0.74^a^	5.76^a^
Website	4.40	0.93^a^	5.55
Facebook Ad	4.68	–0.21^b,c,d^	5.06^b,d^
Electronic Flyer	4.76	0.58^c^	5.72^c^

^a^Facebook.

^b^Mobile app.

^c^Website.

^d^Electronic flyer.

**Table 3:** Pre-post change in likelihood to participate in the clinical trial, stratified by baseline likelihood.

	Pre-exposure likelihood to participate in clinical trial (1-10 Scale)			
	≤2	3-4	5-6	≥7
App	1.65	0.63	0.52	0.11
Website	1.76	1.41	0.54	–0.44
Facebook ad	1.14	0.00	–0.44	–1.63
Electronic flier	1.78	1.55	0.26	–1.67

## Discussion

### Principal Findings

In this study, we generated insights into key challenges and barriers that impede young adult populations from more actively participating in clinical trials. Our social listening study examining Quora identified 763 user-generated questions and 2548 answers that included topics about COVID-19 trials including (1) questioning whether clinical trial results could be trusted, (2) concerns about vaccine safety and efficacy, (3) questions about vaccine trial design and vaccine platform, and (4) discussion about specific barriers to participating in clinical trials. Topics identified as participation barriers included concerns about the safety of trial participation, lack of knowledge on how to participate, and questions about vaccine access if they withdrew from a trial [21]. These results informed the design of our focus group discussions with young adults and themes explored in our co-design sessions.

After co-designing 2 digital tools consisting of a mobile app wireframe and a website with this population, our intercept study found that at baseline most respondents were moderately interested in participating in a clinical trial (4.40-4.76) but after brief exposure to the co-designed digital tools, both the mobile app and website had positive change associated with likelihood to participate and learning more about clinical trials. Though the electronic flier control had comparable results, we observed different levels of change in likelihood to participate for mean change scores, with the mobile app maintaining positive mean change among all users at baseline (1-10 responses), whereas the flier saw negative change for those at high baseline of likelihood (7+). These conclusions generally support the observation that digital tools may be more encouraging for users who have a lower initial interest in trial participation.

Overall, we observed that there was a negative effect on participation likelihood in the group with the highest baseline likelihood, though the app and website were least likely to detract from likelihood in this subgroup, a finding that requires further research and consideration in recruitment technology development. This may indicate that different recruitment modalities (eg, website, mobile app, Facebook ad, and digital flier) have different levels of effectiveness depending on the initial interest of users to participate in a clinical trial and that recruitment tools and strategies need to be customized to specific user characteristics such as baseline interest in trial participation, health literacy measures, and specific trial opportunity characteristics of interest to young adults.

Further, we observed that the mobile app and website had longer periods of exposure based on time spent and engagement time data from our tracking software, likely reflected by the fact that these tools had more options for interaction and engagement (eg, interactivity and customizability of searches) than traditional recruitment methods used in our control exposures. Hence, co-designed digital tools may have the potential for greater information exposure and create a sense of agency among young adults, while offering broader accessibility options such as mobile apps and websites that can be readily downloaded or viewed on the Internet for those interested in exploring clinical trial engagement.

Importantly, co-design sessions with a group of young adults were an approach that enabled the development of user-centered solutions by engaging and empowering end users in the design process. While the research team was responsible for translating co-design session discussions and feedback into tangible prototypes, participants drove the design process and features of the end products. Many co-design participants characterized the tool as a “dating app” or Yelp (eg, crowdsourced business review site) for clinical trials, and continually provided input that layered features, which simplified the purpose and mission of trials in a transparent manner, identified key trial features that could be filtered and matched, and heightened interactivity (eg, notifications, risk information, ability to “follow” a trial for updates). In addition, respondents provided detailed information on expected incentives for participation, including disclosure of trial incentives (eg, compensation) and other suggested microincentives (eg, digital badge, and points on their profile page). These features could not have been developed by the research team alone, demonstrating the promise and potential of a co-design approach in the creation of health and medical innovations such as clinical trial recruitment or enrollment tools [10,22-24].

### Limitations

This study has certain limitations. Based on findings from a previous social listening study, our analysis focused on a single platform where active discussions about trial participation were observed. In addition, the data collection was limited to English keywords, which may impact the generalizability of the results. Focus groups were limited to young adults in one geographic region (Los Angeles-Long Beach-Anaheim Metropolitan Statistical Area) and may limit the generalizability of findings to other regions or cultural contexts. Future studies should include the attitudes and experiences of young adults from other geographic areas. For the intercept study, blinding of participants was not possible, nor was it possible to control communication about the study outside of the experimental setting, which could have led to bias in results. Participants were largely from one ethnic group and should be replicated with greater representation of other racial and ethnic minority young adult populations. In addition, exposures used in the intercept study for control and intervention groups were meant to simulate traditional forms of trial recruitment materials (eg, recruitment fliers and mock Facebook recruitment ads) and compare these traditional approaches to digital tools (eg, co-designed mobile and internet-based recruitment tools). However, controls and interventions are inherently different in their presentation and modalities of user engagement which may impact the interpretability of results. The general absence of free and publicly available digital health recruitment tools limited our ability to use more similar comparative controls and intervention exposures. Importantly, this study measured proxies for enrollment via interest and intentionality but did not measure differences between arms for the downstream behavior itself. A future study should further validate these results by simulating enrollment in clinical trials or testing the tool for recruitment into real-world trials.

### Conclusions

In summary, goals outlined by a 2022 report by the National Academies of Sciences, and Engineering about building trust and promoting fairness for clinical trial participation are themes that were echoed in our study [25]. Patient-centered approaches that involve the active participation of diverse young adults have the potential to directly address these goals, while also building these needs and values directly into their technology design, though additional work and iteration are needed in alignment with principles of digital health to bring such a solution to market [12,26].

## Appendix

Multimedia Appendix 1 Additional web-based content.

## Abbreviations

FAQ: frequently asked question
IRB: institutional review board
MSI: Minority Serving Institution
RA: research assistant

## Data Availability

The deidentified datasets generated and analyzed during this study are available from the corresponding author on reasonable request.
