# Supplementary material for: Approach to Design and Evaluate Digital Tools to Enhance Young Adult Participation in Clinical Trials: Co-Design and Controlled Intercept Study
Source: J Med Internet Res. 2025 Apr 11;27:e70852. doi: 10.2196/70852 (PMC12032498; doi:10.2196/70852)
Supplement: Multimedia Appendix 1 [file jmir_v27i1e70852_app1.docx]

**Supplemental Online Content**

**eMethods.**

**eReference**

This supplemental material has been provided by the authors to give readers additional information about their work.

eMethods.

eSupplement Appendix

**Intercept Study Details:**

User time spent on the mobile application, website, mock clinical trial recruitment flier, and mock Facebook trial recruitment ad were recorded using Hotjar (mobile app) and Mouseflow (for website, flier, Facebook ad). Engagement time was generated by Mouseflow for the website, flier, and Facebook ad but not for the mobile app as this metric was not available from Hotjar. Engagement time is described as the average engagement time that accounts for the duration of user activity minus inactivity for a website/page. Examples of aggregated heat maps of user interaction with mobile app and website are provided in Supplementary Figures 1-4.

Supplementary Figure 1: User interactivity heat map for mobile app


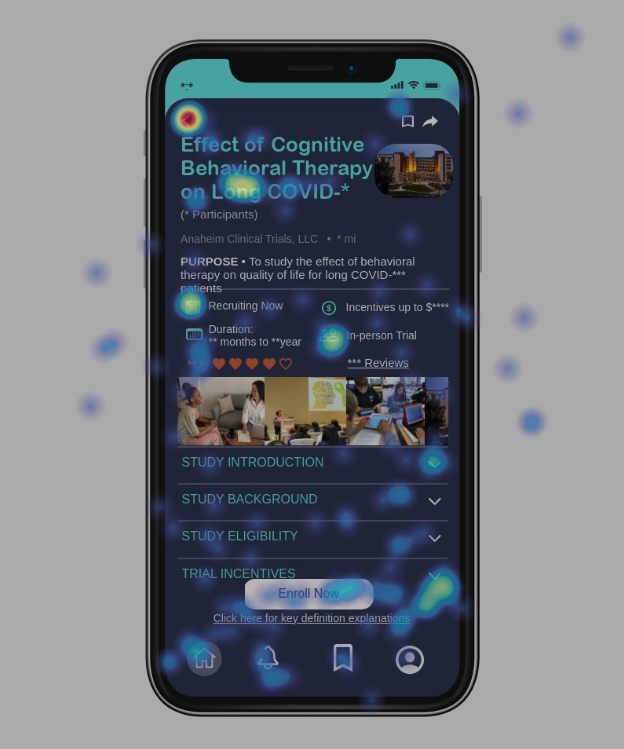


Supplementary Figure 2: User interactivity heat map for website


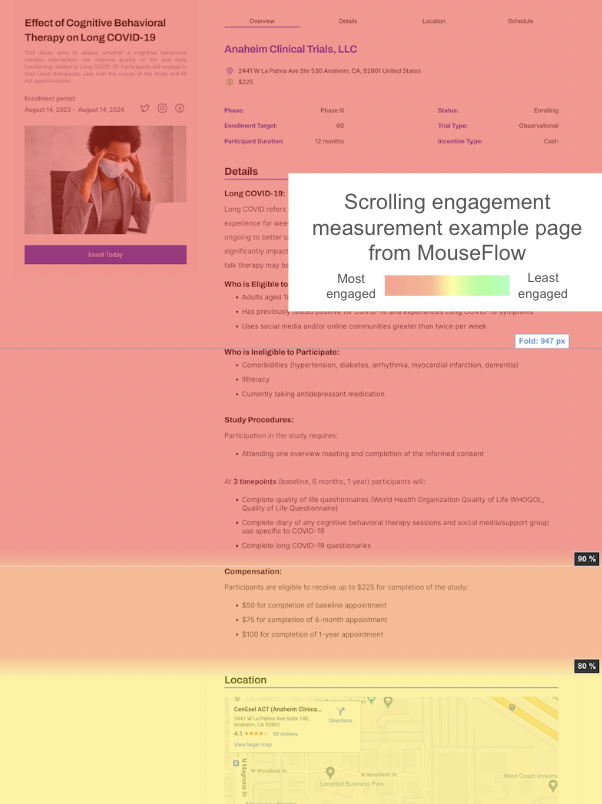


Supplementary Figure 3: User interactivity heat map for mock flier


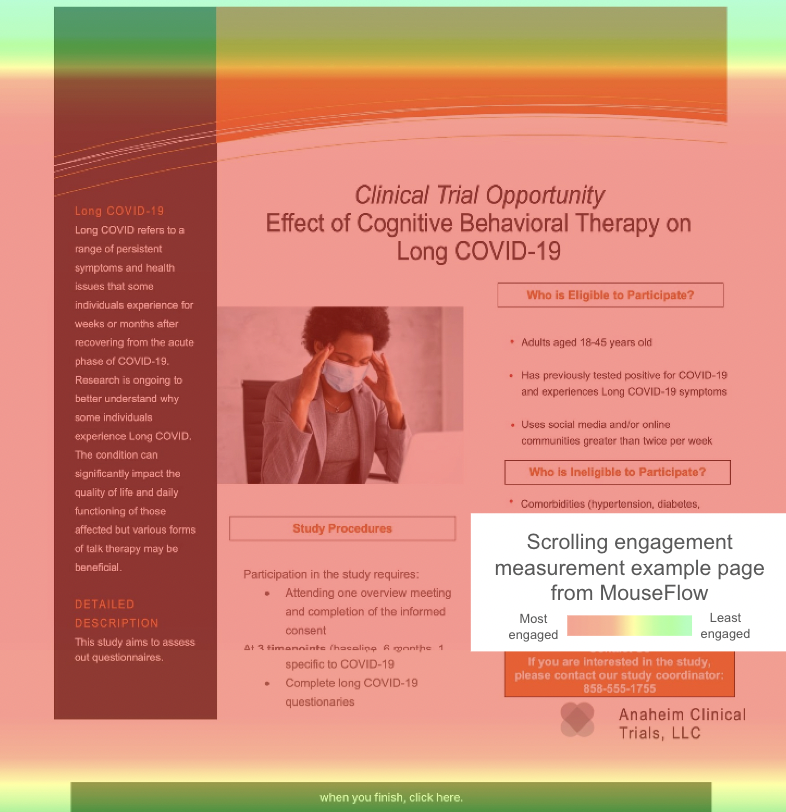


Supplementary Figure 4: User interactivity heat map for mock Facebook ad


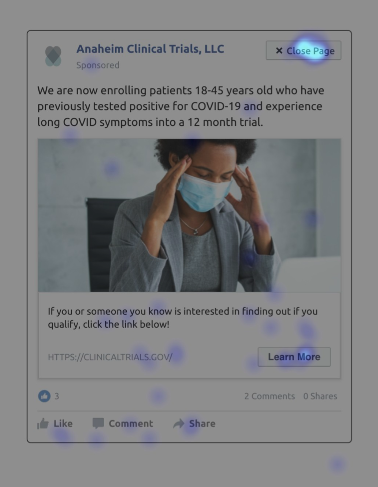


**Supplement Documentation. Intercept Questionnaire.**

Intercept Survey

Welcome and thank you for participating in this study! Your input will help us understand factors that are important in enrolling people in clinical trials. If you have previously participated in this study, please inform the research liaison who asked you to participate.

Please click “Next page” to read the informed consent form.

**CALIFORNIA STATE UNIVERSITY, FULLERTON**
**RESEARCH STUDY CONSENT FORM**
**PHASE 3**

**Study Title:** Co-creation of digital tools to enhance young adult minority participation in COVID-19 trials

**Protocol Number:** HSR-21-22-309

**Researchers**:             Joshua Yang, Ph.D., Department of Public Health, California State University, Fullerton (Principal Investigator)

**Sponsor**:                   U.S. Food and Drug Administration

You are being asked to take part in a research study carried out by Dr. Joshua Yang (Principal Investigator), Professor, California State University, Fullerton (CSUF). This consent form explains the research study and your part in it if you decide to join the study. Please read the form carefully, taking as much time as you need. Please ask the researcher to explain anything that you do not understand.

 Participation in this study is **completely voluntary**. You can decide not to join the study. If you join the study, you can change your mind later and leave the study at any time. There will be no penalty or loss of services or benefits if you decide not to participate in the study.

**What is this study about?**
This research study is being conducted to assess the effectiveness of a COVID-19 clinical trials informational tool. You are being asked to take part because you meet the following qualifications:

 (1) age 18 to 29 years old.

Taking part in the study will take about five minutes. You cannot take part in this study if you are under 18 years old or do not meet other study criteria listed above.

**What will I be asked to do if I am in this study?**
If you volunteer to take part in this study, you will be asked to answer questions about your interest in participating in a clinical trial. Then, you will be asked to view clinical trials materials and answer some demographic questions and some additional questions about the likelihood of participation in a clinical trial. Overall, your participation will take 5 minutes.  

**Are there any benefits to me if I am in this study?**
There are no direct benefits to you from being in this study. However, by participating in the study, you might gain increased knowledge of clinical trials for COVID-19.

**Are there any risks to me if I am in this study?**
The potential risks from taking part in this study are the time you spend viewing the study material and completing the questionnaires. In order to protect against this risk, you may terminate your participation at any time.

**Will my information be kept anonymous and confidential?**
The data for this study will be kept confidential to the extent allowed by law and reasonable ethical standards. Under certain circumstances, information that identifies you may be released for internal and external reviews of this project. No published results will identify you, and your name will not be associated with the findings.

Your data will be protected in the following ways:

 1. Your name and contact information you submit to be part of an opportunity drawing will be separated from other information you enter as part of the study once the data are downloaded and will only be used to select winners of the opportunity drawing.
 2. All study data will be stored on ID- and password-protected computers and in locked facilities.
 3. The results of this study may be published or presented at professional meetings, but the identities of all research participants will not be used, and no other potentially identifying information will be included.

Data will be accessible only to the principal investigator and research staff.
              
The data for this study will be kept indefinitely. The data will be used for future presentations, publications, and to inform efforts to increase young adult participation in clinical trials.

**Are there any costs or payments for being in this study?**
If you take part in the study, you will be entered into an opportunity drawing to receive one of 5 $50 gift card at the end of the study. In order to be eligible to receive the gift card, you must provide either your email address or  a mailing address.

**Who can I talk to if I have questions?**
If you have questions about this study or the information in this form, please contact the Principal Investigator, Dr. Joshua Yang, by email at jsyang@fullerton.edu, or by phone at (657) 278-4384.

If you have questions about your rights as a research participant, or would like to report a concern or complaint about this study, please contact the Institutional Review Board at (657) 278-7719, or e-mail irb@fullerton.edu.

**What are my rights as a research study volunteer?**
Your participation in this research study is completely voluntary. You may choose not to be a part of this study. There will be no penalty to you if you choose not to take part. You may choose not to answer specific questions or to stop participating at any time.

**What does entering my name on this consent form mean?**
Your signature on this form means that: You understand the information given to you in this form; You have been able to ask the researcher questions and state any concerns; The researcher has responded to your questions and concerns; You believe you understand the research study and the potential benefits and risks that are involved.

**Statement of Consent**
I have carefully read the terms used in this consent form.  By entering my name below, I agree that I am at least 18 years of age and agree to participate in this project.  A copy of this informed consent will be provided to you upon request.

- Name: __________________________________________________
- Date: __________________________________________________

By entering your name above and clicking the "Next Page" button, you will be agreeing to participate in this study.

Thank you for agreeing to participate. Please answer the following question honestly and to the best of your ability.

At this time, how likely are you to participate in a clinical trial?

|  | Not Likely | Moderately Likely | Very Likely |
| --- | --- | --- | --- |

|  | 1 | 2 | 3 | 4 | 5 | 6 | 6 | 7 | 8 | 9 | 10 |
| --- | --- | --- | --- | --- | --- | --- | --- | --- | --- | --- | --- |

|  | 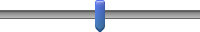 |
| --- | --- |

Please click on the link below to continue with this survey. It will take you to a new window. When you are finished viewing the clinical trial material in the new window, close it, return to this page and click "Next page"

 https://trial-match.webflow.io/flyer-1

Please click on the link below to continue with this survey. It will take you to a new window. When you are finished viewing the clinical trial material in the new window, close it, return to this page and click "Next page"

 https://trial-match.webflow.io/flyer-2

Please click on the link below to continue with this survey. It will take you to a new window. When you are finished viewing the clinical trial material in the new window, close it, return to this page and click "Next page"

 https://trial-match.webflow.io/flyer-3

Please click on the link below to continue with this survey. It will take you to a new window. When you are finished viewing the clinical trial material in the new window, close it, return to this page and click "Next page"

 https://trial-match.webflow.io/facebook-ad-2

Please click on the link below to continue with this survey. It will take you to a new window. When you are finished viewing the clinical trial material in the new window, close it, return to this page and click "Next page"

 https://trial-match.webflow.io/facebook-ad-1

Please click on the link below to continue with this survey. It will take you to a new window. When you are finished viewing the clinical trial material in the new window, close it, return to this page and click "Next page"

 https://trial-match.webflow.io/facebook-ad-3

Please click on the link below to continue with this survey. It will take you to a new window. When you are finished viewing the clinical trial material in the new window, close it, return to this page and click "Next page"

The clinical trial material you are about to view is a mobile application in beta version. As you navigate the mobile app, please note that the mobile application may have limited functionality for certain features and has a preset user profile.

 https://cloud.justinmind.com/usernote/prototype/f3c78d6c61f286bae0256f8b15d98be1cd5e4dc3ff55a13e8de2f440378ce086

Please click on the link below to continue with this survey. It will take you to a new window. When you are finished viewing the clinical trial material in the new window, close it, return to this page and click "Next page"

The clinical trial material you are about to view is a website in beta version. As you navigate the website, please note that this website may have limited functionality for certain features, buttons, or commands.

 https://trial-match.webflow.io/

Thank you for interacting with the clinical trial material in the previous window. Please answer the following questions based on your experience.

After viewing the material in the previous window, how likely are you now to participate in a clinical trial?

|  | Not Likely | Somewhat Likely | Very Likely |
| --- | --- | --- | --- |

|  | 1 | 2 | 3 | 4 | 5 | 6 | 6 | 7 | 8 | 9 | 10 |
| --- | --- | --- | --- | --- | --- | --- | --- | --- | --- | --- | --- |

|  | 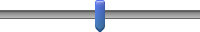 |
| --- | --- |

After viewing the material in the previous window, how much more interested are you in learning about clinical trials?

|  | No More Interested | Somewhat More Interested | Much More Interested |
| --- | --- | --- | --- |

|  | 1 | 2 | 3 | 4 | 5 | 6 | 6 | 7 | 8 | 9 | 10 |
| --- | --- | --- | --- | --- | --- | --- | --- | --- | --- | --- | --- |

|  | 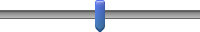 |
| --- | --- |

What is your age?

▼ 18 ... Prefer not to answer

Are you Hispanic or Latino?

- Yes, Hispanic or Latino
- No, not Hispanic or Latino
- Prefer not to say

What is your race? Select one or more

- American Indian or Alaska Native
- Asian
- Black or African American
- Native Hawaiian or Other Pacific Islander
- White
- I use a different term: __________________________________________________
- Prefer not to answer

| Page Break |  |
| --- | --- |

What sex were you assigned at birth, on your original birth certificate?

- Female
- Male
- Don't know
- Prefer not to answer

What is your current gender?

- Female
- Male
- Transgender
- Two-Spirit
- I use a different term: __________________________________________________
- Don't know
- Prefer not to answer

Which of the following best represents how you think of yourself?

- Lesbian or gay
- Straight, that is, not gay or lesbian
- Bisexual
- Two-Spirit
- I use a different term: __________________________________________________
- Don’t know
- Prefer not to answer

Have you ever been diagnosed by a medical doctor or other health professional with an intersex condition or a difference of sex development (DSD) or were you born with (or developed naturally in puberty) genitals, reproductive organs, or chromosomal patterns that do not fit standard definitions of male or female?

- Yes
- No
- Don't know
- Prefer not to answer

What is your major?

▼ Accountancy ... Prefer not to say

What is the highest level of education completed by either of your parents?

- Less than high school
- High school graduate
- Some college
- 2 year degree (e.g. AS, AA)
- 4 year degree (e.g. BS, BA)
- Master's degree (e.g. MS, MA)
- Professional degree (e.g. MD, DO, DDS)
- Doctorate (e.g. PhD, ScD)
- Don't know
- Prefer not to answer

What is your estimated annual family household income?

- Less than $25,000
- $25,000 - $49,999
- $50,000 - $74,999
- $75,000 - $99,999
- $100,000 - $149,999
- $150,000 or more
- Prefer not to answer

You have the option of entering an opportunity drawing for a chance to win one of five $50 gift cards. If you would like to participate, please provide your name and contact information below.

Name:

________________________________________________________________

Contact Information (phone number or email address):

________________________________________________________________

Thank you for your participation in this study! Your input is valuable in helping us improve participation and achieving diversity in clinical trials.
